# Les traumatismes urétéraux compliquant une chirurgie gynécologique

**DOI:** 10.11604/pamj.2018.30.145.15470

**Published:** 2018-06-20

**Authors:** Fatnassi Ridha, Merzougui Latifa, Rebhi Ines, Hajji Maamar, Braiek Salem

**Affiliations:** 1 Service de Gynécologie, CHU Ibn El Jazzar, Kairouan, Tunisie; 2 Service d’Hygiène Hospitalière, CHU Ibn El Jazzar, Kairouan, Tunisie; 3 Service d’Urologie, CHU Ibn El Jazzar, Kairouan, Tunisie

**Keywords:** Lésion urétérale iatrogène, ligature urétérale, hystérectomie, réimplantation urétéro-vésicale, Iatrogenic ureteral lesions, ureteral ligation, hysterectomy, uretero-vesical reimplantation

## Abstract

Les lésions urétérales iatrogènes peuvent succéder à toute chirurgie pelvienne. Elles sont graves pouvant mettre en jeu le pronostic fonctionnel du rein et même le pronostic vital. But du travail: est de préciser les aspects cliniques et les modalités thérapeutiques de cet accident. Il s’agit d’une étude rétrospective sur six observations de lésion urétérale iatrogène, après chirurgie gynécologique ou obstétricale, colligées dans le service d’urologie de Kairouan sur une période de 4 ans (2012-2016). L’âge moyen de nos patientes est de 46 ans. Elles sont toutes des multipares. La symptomatologie clinique est variable selon le type lésionnel. L’urographie intraveineuse demeure très utile au diagnostic, nous l’avons pratiqué dans 4 cas revenues toutes anormales. Le traitement a consisté en une montée de sonde dans un cas et 5 réimplantations urétéro-vésicales. Les suites opératoires sont émaillées par une néphrectomie. Les lésions urétérales iatrogènes sont devenues rares. Elles sont corrélées au degré de médicalisation du pays. La chirurgie gynécologique ou obstétricale est la plus grande pourvoyeuse. Leur pronostic est conditionné par la précocité du diagnostic et l’état anatomique de l’uretère.

## Abstract (en)

Iatrogenic ureteral lesions may occur after any pelvic surgery. They are severe and can affect renal function and even vital prognosis. This study aimed to determine the clinical aspects and the therapeutic approaches of this injury. We conducted a retrospective study of six patients with iatrogenic ureteral lesions following gynecologic or obstetric surgery whose data were collected in the Department of Urology in Kairouan on a period of 4 years (2012-2016). The average age of our patients was 46 years. They were all multiparous. Clinical symptoms were variable depending on the type of lesion. Intravenous urography is very useful for diagnosis. It was performed in 4 cases showing abnormalities. Treatment was based on the insertion of a catheter in one case and on 5 uretero-vesical reimplantations. The postoperative course was marked by a nephrectomy. Iatrogenic ureteral lesions have become a rarity. They are correlated with the degree of medicalization in the country. Gynecological and obstetric surgery is the main cause of iatrogenic ureteral lesions. Prognosis is conditioned by early diagnosis and the anatomic condition of the ureter.

## Introduction

La chirurgie pelvienne représente une véritable menace pour l’uretère, en effet la portion pelvienne de l’uretère est exposée au risque de lésion tout au long de son trajet. Il s’agit le plus souvent de lésions iatrogènes secondaires particulièrement à la chirurgie gynécologique et obstétricale [1]. Cette vulnérabilité est la conséquence des rapports intimes qu’entretient cet organe avec l’appareil génital chez la femme. La pathologie secondaire à ces lésions constitue une véritable infirmité pour la malade, une menace pour le rein sus jacent et un embarras pour le chirurgien qui doit la traiter. A travers six observations de patientes ayant présenté des lésions urétérales nous allons tacher d’étudier les aspects étiologiques, et cliniques de ces accidents ainsi que ses modalités thérapeutiques.

## Méthodes

Il s’agit d’une étude rétrospective menée au service d’urologie de Kairouan et réalisée sur une période de 4 ans (février 2012- février 2016). Elle est basée sur l’étude des dossiers. Les éléments étudiés sont: l’âge et la parité des patientes, le motif de consultation, le type d’intervention initiale, le délai écoulé avant le diagnostic, les éléments du diagnostic et la conduite thérapeutique. Durant cette période nous avons colligé 6 observations de plaies urétérales iatrogènes, secondaires à une chirurgie gynécologique et obstétricale.

## Résultats

L’âge moyen de nos patientes est de 46 ans avec des extrêmes de 36 à 60 ans. Toutes nos patientes sont des multipares avec une parité qui varie de 3 à 12. Par ailleurs, elles n’ont pas d’antécédents médicaux ou chirurgicaux notables. La chirurgie gynécologique et obstétricale a constitué l’unique pourvoyeuse de lésions urétérales dans notre série. Elle varie de la kystectomie simple à l’hystérectomie élargie, la césarienne était en cause dans un cas. Le traumatisme urétéral est bilatéral dans un cas et unilatéral du côté droit dans 5 cas ; il s’agit d’une ligature section urétérale dans 3 cas et d’une ligature urétérale dans deux cas (Tableau 1). Le diagnostic de la lésion urétérale a été posé en post opératoire dans tous les cas, avec un délai variable de 1 à 13 jours. Aucune atteinte urétérale n’a été diagnostiquée en per opératoire. Les circonstances de découverte sont polymorphes et varient selon le type lésionnel: 1) La fuite urinaire permanente par le vagin = 2 cas; 2) La fuite urinaire par le drain de redon = 1 cas; 3) Les douleurs lombaires = 2 cas; 4) L’anurie totale par ligature urétérale bilatérale = 1 cas. L’examen physique a révélé la fuite d’urine par le vagin dans deux cas et l’épreuve au bleu de méthylène était positive dans un cas, il s’agit d’une fistule vésico-vaginale associée à la lésion urétérale (Tableau 2).

**Tableau 1:** Caractéristiques des patientes

Patiente	Age (ans)	Gestité/parité	*Chirurgie initiale*		*lésion urétérale*	
			*Intervention*	*indication*	*Nature de lésion*	*Coté*
1	45	5/5	Césarienne	Défaut d'engagement	Ligature section	Droit.
2	45	8/8	Hystérectomie totale par voie haute	Ménométrorragies	Ligature	Droit
3	60	11/13	Hystérectomie totale avec annescectomie bilatérale	Tumeur ovarienne	Ligature	Bilatéral
4	46	11/11	Hystérectomie totale par voie haute	Utérus fibromateux	Ligature section * Fistule vesico vaginale associée.	Droit
5	40	3/4	Hystérectomie d'hémostase	Rupture utérine	Ligature	Droit.
6	40	7/12	kystectomie	Kyste hydratique pelvien	Ligature section	Droit.

**Tableau 2:** Aspects cliniques et thérapeutiques des plaies urétérales

Patientes	Délai diagnostic (jour)	Circonstances de diagnostic	Eléments du diagnostic	Type de traitement	Séjour	suites opératoires
1	10	Fuite urinaire par le vagin	**UIV:** dilatation urétéropyélocalicielle droite en amont d'un obstacle urétéral pelvien	RUV* bilatérale type leadbetter politano	12 j	favorable
2	1	Colique néphrétique droite	**UIV:**obstruction de l'uretère pelvien droit.	Montée de sonde urétérale	14 j	bonne
3	1	anurie	**Echographie:** dilatation urétéropyélocalicielle bilatérale	RUV* bilatérale type leadbetter politano	10 j	favorable
4	13	Fuite urinaire par le vagin	**UIV:** retard d'excrétion du rein droit avec dilatation urétéropyélocalicielle droite.	RUV* type Boari-Kuss	15 j	phlébite du MI. néphrectomie
5	5	Douleur lombaire droite	**Echographie :** urétérohydronéphrose du rein droit.	RUV* bilatérale type leadbetter politano	11 j	favorable
6	4	Fuite urinaire par le drain de redon	**Uroscanner :** dilatation de l’uretère pelvien droit avec extravasation du produit de contrast.	RUV* bilatérale type leadbetter politano	10 j	favorable

* RUV : réimplantation urétéro-vésicale

**Figure 1:**
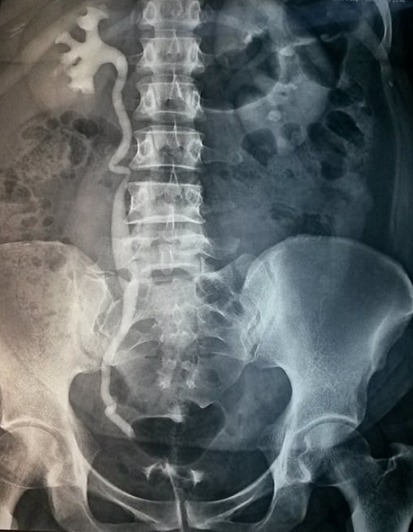
Dilatation urétéro-pyélo-calicielle droite en amant d’un obstacle pelvien et absence d’opacification à gauche secondaire à une ligature urétérale complète après hystérectomie

**Figure 2:**
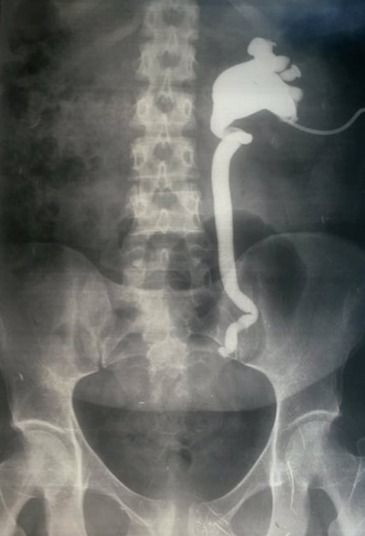
Nephrostomie percutanée avec opacification montrant une dilatation urétéro-pyélo-calicielle en amant de la ligature urétérale gauche

**Figure 3:**
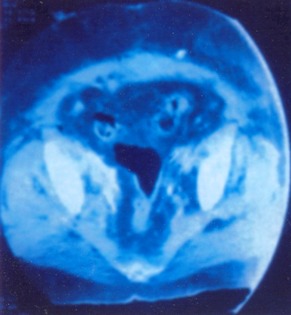
Fuite de produit de contraste pelvienne droite

Les explorations para-cliniques ont comporté une urographie intra-veineuse (U.I.V) dans 4 cas, qui a montré une dilatation urétéropyélocalicielle droite dans trois cas et une dilatation bilatérale dans un cas (Figure 1, Figure 2). Dans un cas l’uro-scanner avec des clichés tardifs,pratiqué chez une patiente retrouve un urinome (Figure 3). Le traitement a été chirurgical et a fait l’objet de techniques variées. Il a consisté en une montée de sonde urétérale dans un cas avec évolution favorable et 4 réimplantations urétéro-vésicales types politano-lead better dont une bilatérale avec vessie psoïque dans un cas, et une réimplantation avec lambeau vésical selon la technique Boari Kuss dans un autre cas. Les suites opératoires ont été favorables dans 4 cas avec un séjour hospitalier moyen de 12 jours (10 à 15jours). La patiente qui a bénéficié d’une réimplantation type Boari Kuss, a développé une thrombophlébite du membre inférieur avec persistance des fuites urinaires. L’U.I.V de contrôle trouve un rein droit peu fonctionnel et très dilaté. Une néphrectomie a été réalisée avec des suites favorables (Tableau 2).

## Discussion

L’estimation globale de la fréquence des lésions opératoires de l’uretère est très variable suivant les séries analysées allant de 0.5 à 30% [2, 3]. En fait cette fréquence dépend de plusieurs facteurs. En effet: 1) Le degré de médicalisation joue un rôle très important et les complications urinaires iatrogènes d’origine obstétricale sont surtout l’apanage de pays en voie de développement [4, 5]; 2) Le type de chirurgie effectuée (Tableau 3): qu’il s’agisse de chirurgie par voie abdominale ou vaginale, l’uretère est toujours menacé [1, 6-8] ainsi Symmonds retrouve 2 à 3% de lésions urétérales après hystérectomie tandis que Robert rapporte 3,6% [9]. Les hystérectomies élargies avec lymphadenectomie (type Wertheim) sont les plus grandes pourvoyeuses de lésions urétérales: 10 à 30% des cas. Cependant, la chirurgie coelioscopique n’épargne pas cet organe; Chapron rapporte un taux de 1,^7^% des plaies urétérales au cours de la coeliochirurgie [10].

**Tableau 3:** Etiologies des lésions urétérales selon les séries

*Etiologies*	*Séries*				
	Française (4)	Marocaine (7)	Tunisienne (5)	Benin (6)	Notre série
**CAUSES GYNECOLOGIQUES**	40	13	11	3	3
***Hystérectomie abdominale***					
Fibrome utérin	23				
Cancer du col		3	3	2	
Cancer de l'endomètre		8	3		1
Prolapsus	3	2		1	
***Hystérectomie vaginale***					
Fibrome utérin	2		3		
Prolapsus	2		2		
Mraschall-marechetti	3				
Tumeur de l'ovaire	6				1
Kystectomie	1				1
**CAUSES OBSTETRICALES**	7	29	19	5	3
***Manœuvre obstétricale***					0
Forceps		3	3		0
Aspiration endo-utérine	1				
***Chirurgie obstétricale***					
Hystérorraphie pour rupture utérine		5	4		
Hystérectomie pour rupture utérine.		4	3	2	1
Hystérectomie d'hémostase	2		3		1
Césarienne	4	17	6	3	1

Dans les pays en voie de développement c’est surtout les femmes jeunes et primipares qui sont intéressées par les plaies urétérales d’origine gynéco-obstétricale [7], par contre dans les pays développés ces lésions sont l’apanage des multipares. Dans notre série, toutes les femmes sont multipares. La précocité du diagnostic conditionne le résultat thérapeutique [11, 12]: 1) L’idéal serait de reconnaître cet accident en per opératoire mais ce fait est rare: pour Cussenot O [13], le diagnostic a été porté en per opératoire dans 20% des cas et pour Benoit G [14] dans 10 % des cas. Dans la série tunisienne le diagnostic per opératoire des plaies urétérales n’a pu être fait que dans 7.5% des cas [6]; 2) Dans la majorité des cas, ces lésions sont reconnues en post opératoire ; ainsi pour El Ouakdi [6] le diagnostic a été établi au delà du 1^er^ mois dans 55 % des cas et au cours du 1er mois dans 37.5% des cas. Dans notre série le diagnostic des lésions a été fait au cours des 10 premiers jours (Tableau 4).

**Tableau 4:** Le délai diagnostic selon les séries

Délai	Série			
	Tunisienne (5)	Marocaine (7)	Française (4)	Notre série
Per-Opératoire	2	8	4	-
S < 1 Moi	10	32	21	6
Entre 1moi et 1 an	18	2	7	-
> 1 an	-	-	15	-

Les signes révélateurs sont essentiellement les fuites urinaires et les douleurs lombaires, rarement l’accident est révélé par une anurie ou par une masse pelvienne (témoignant d’un urinome) ou par hématurie. Dans différentes séries [6], la fuite urinaire par le vagin représente le signe révélateur le plus fréquent, suivie par les douleurs lombaires. Notre série bien que courte retrouve les mêmes constatations. Si les signes révélateurs sont généralement très bruyants, l’examen clinique est par contre pauvre en dehors de la constatation d’un écoulement d’urines par le vagin, par le système de drainage ou par la plaie opératoire. La palpation recherche un empâtement pelvien témoin d’épanchement urineux. L’examen vaginal révèle, en cas de fuite urinaire, l’orifice externe de la fistule et recherche une éventuelle fistule vésico-vaginale associée. L’épreuve au bleu de méthylène confirme l’intégrité de la vessie sauf s’il y a une fistule vésico-vaginale associée. Les examens complémentaires vont d’une part permettre de préciser le diagnostic évoqué et d’autre part apprécier la fonction et l’intégrité de l’appareil urinaire controlatéral. L’échographie, examen non invasif est devenue l’exploration de première intention: elle montre une dilatation des voies excrétrices et donne une idée sur l’index parenchymateux; elle est particulièrement utile en cas de rein muet à l’UIV [15]. Pratiquée chez 5 de nos patientes, elle s’est révélée anormale dans 2 cas retrouvant une dilatation des voies excrétrices.

L’UIV était l’examen essentiel pour établir le diagnostic positif des lésions urétérales [16]. Elle permet: 1) De préciser le retentissement du traumatisme urétéral sur le haut appareil urinaire; 2) D’apprécier l’état du rein controlatéral; 3) De préciser le siège du traumatisme urétéral Cet examen a été pratiqué chez 4 de nos patientes et s’est révélée anormale dans tous les cas. La nephrostomie avec opacification permet en cas d’impossibilité de cathétérisme rétrograde de visualiser l’uretère. Actuellement l’uroscanner est l’examen de choix pour mettre en évidence la fuite urinaire et localiser son siège [11, 17]. On a eu recours à cette exploration chez une patiente qui avait une fuite par le système de drainage et chez laquelle l’UIV n’a pas montré de produit de contraste. l’uroscanner à permis de mettre en évidence la fuite de produit de contraste au niveau de l’uretère pelvien (Figure 2) elle peut avoir aussi un intérêt dans le diagnostic de pathologie causale. L’inconvénient majeur de cet examen son taux d’irradiation. L’imagerie par résonnance magnétique nucléaire (IRM) présente l’avantage d’être non irradiante et d’avoir une meilleure résolution en contraste. L’examen comporte des séquences en pondération T1, T2, et T1 après injection de gadolinium [17]. La sensibilité de l’IRM dans le diagnostic des lésions urétérales iatrogènes est de l’ordre de 86,8% [18].

La recherche d’une infection urinaire et l’appréciation de la fonction rénale seront systématiques dans ces situations. Le traitement aura pour objectif de rétablir la continuité urétérale et dépendra de plusieurs paramètres: 1) Les données anatomiques : état de l’uretère (surtout la portion distale), état du rein, de la vessie et les lésions associées; 2) L’état de la malade: âge, état général, le type de la maladie initiale ayant conduit à l’intervention responsable de la lésion; 3) Les moyens techniques à la disposition du chirurgien et sa compétence; 4) Les méthodes endoscopiques permettent souvent une approche simple de la pathologie et ont un rôle diagnostique et thérapeutique. Le cathétérisme urétéral rétrograde représente la première initiative. En cas d’échec, on a recours à la voie per cutané avec cathétérisme antérograde de la voie excrétrice [9, 19]; 5) La chirurgie à ciel ouvert est indiquée en cas d’échec du traitement endourologique.

Le traitement chirurgical va consister soit à une simple réparation de l’organe lésé soit à une réimplantation urétérovésicale éventuellement associée à des artifices plus ou moins complexes [20-22]. Dans notre série, trois patientes ont bénéficiées d’une montée de sonde, un cas a été couronné de succès avec guérison totale et dans deux cas, la sonde bute contre un obstacle. On a eu recours à une réimplantation urétéro vésicale dans 5 cas. Dans la littérature on retrouve un succès du traitement endourologique dans 38 à 88% des cas [23], tandis que dans notre série on a eu une seule guérison, en fait, les succès de ce traitement dépendent essentiellement du caractère de la lésion autorisant ou non le passage de la sonde. Le pronostic dépend des conditions anatomiques (état de l’uretère et lésions associées) du délai de prise en charge, de l’état général de la femme et de l’expérience du chirurgien. Dans notre série, il y a eu guérison dans tous les cas avec cependant la perte d’un rein dans un cas.

## Conclusion

Les conséquences des lésions urétérales sont lourdes et tout chirurgien abordant le pelvis devrait avoir à l’esprit ce risque car l’appareil urinaire et l’appareil génital sont intimement liés depuis le stade embryonnaire. La connaissance de ce risque devrait inciter à plus de prudence surtout lorsque les rapports anatomiques du pelvis sont modifiés par un processus inflammatoire, tumoral ou une endométriose. La pratique d’une montée de sonde en préopératoire en cas de chirurgie à haut risque reste la meilleure sagesse. Le diagnostic précoce est basé sur une surveillance post opératoire régulièrement: dépistage des signes fonctionnels, et au moindre doute un examen échographique.

### Etat des connaissances actuelles sur le sujet

- La chirurgie pelvienne est à haut risque de lésions urétérales;
- Le pronostic est conditionné par la précocité du diagnostic.

### Contribution de notre étude à la connaissance

- Modalités d’un diagnostic précoce;
- L’uro-scanner est actuellement l’examen de référence.

## Conflits d’intérêts

Les auteurs ne déclarent aucun conflit d’intérêts.
